# Beyond the here and now: mood, death attitudes, and diverse future time perspective in late adulthood

**DOI:** 10.3389/fpsyg.2026.1921975

**Published:** 2026-09-23

**Authors:** Celina Timoszyk-Tomczak, Maria Ligocka

**Affiliations:** Institute of Psychology, University of Szczecin, Szczecin, Poland

**Keywords:** death attitudes, future time perspective, late adulthood, transcendent future, transcendental future

## Abstract

**Background:**

Human life is embedded in time, and future time perspective becomes particularly salient in late adulthood. This study examined how three dimensions of death attitudes (contemplation of death, fear of death, belief in life after death) and general mood relate to four dimensions of future orientation in late adulthood: positive future, negative future, transcendental future, and transcendent future, considering age and gender.

**Methods:**

A total of 446 participants aged 60 to 94 years completed the Death Attitude Questionnaire, measures of future time perspective (positive, negative, transcendental, transcendent future), and the General Mood Scale. Correlational analyses, regression-informed path analysis, and MANOVA were conducted.

**Results:**

Lower general mood was associated with higher negative future and higher fear of death, whereas belief in life after death and contemplation of death showed strong links with transcendental and transcendent future. In the path model, negative future was predicted by lower mood and higher fear of death and contemplation of death, while positive future showed positive associations with higher mood and belief in life after death. Transcendental future was strongly predicted by belief in life after death, with positive associations with contemplation of death and fear of death, whereas transcendent future was predicted by belief in life after death and contemplation of death. Women scored higher on transcendental and transcendent future than men.

**Conclusion:**

Future perceptions in late adulthood are closely linked to mood and ways of thinking about death, and associations differ between personal future within this life and future extending beyond one’s own life.

## 1 Introduction

Late adulthood is associated with a shortening of the personal future time horizon, and the future is perceived as more limited than in earlier stages of life (Lang and Carstensen, 2002). Meta-analyses confirm a curvilinear relationship between age and future time perspective: the future horizon is short in youth, expands in mid-adulthood, and then narrows again in old age (Kooij et al., 2018). In youth, the main focus is on optimizing the future, whereas in old age attention shifts toward activities that are meaningful in the present (Charles and Carstensen, 2010). The future nevertheless remains an important temporal dimension. In late adulthood, reflection on death and on time beyond one’s own life becomes more salient, opening up a perspective of transcendence (Timoszyk-Tomczak and Bugajska, 2012, 2019; Zimbardo and Boyd, 2008). Thus, the future can expand to include dimensions that go beyond both the temporal limits of one’s lifetime and personal boundaries (Timoszyk-Tomczak and Bugajska, 2019).

A restricted future time horizon is associated with a range of changes in individuals’ functioning. When the personal future within this life is perceived as limited in late adulthood, psychological well-being tends to be lower and depressive symptoms higher than in situations where the time perspective is more open (Coudin and Lima, 2011). The degree to which the future within this life is constrained, and the way goals are prioritized, influence fluctuations in mood, anxiety, and depression in older adults (Kozik et al., 2015). From a developmental perspective, achieving ego integrity—a sense of coherence, wholeness, and acceptance of one’s unique life—in late adulthood (Erikson and Erikson, 1997) supports a sense of meaning and purpose and is associated with psychological well-being (Hicks et al., 2012).

Research on future orientation in older adults is still relatively scarce, probably because the future is strongly associated with youth. Yet the future in old age, like the broader construct of time perspective, has its own specificity (Timoszyk-Tomczak and Bugajska, 2012). It can be related to a sense of coherence and positive aging, but also to less favorable states (Wiesmann et al., 2018). Studies show that a positive mood becomes more strongly related to a sense of meaning in life as the personal future time horizon becomes constrained (Hicks et al., 2012). In addition, age and gender are important sociodemographic variables that may shape both future time perspective and attitudes toward death (Lang and Carstensen, 2002; Neimeyer et al., 2004). Previous research suggests that women may report greater death-related anxiety, whereas age-related changes in perceived time remaining may influence future orientation and the ways in which individuals engage with mortality (Lang and Carstensen, 2002; Neimeyer et al., 2004). Therefore, examining the links between future time perspective and death attitudes in the context of general mood, age, and gender may help to better understand psychological processes characteristic of late adulthood.

Death is an inherent aspect of human existence (Próchniak, 2005). It is complex and ambiguous (Piotrowski and Żemojtel-Piotrowska, 2004). Awareness of death modifies the hierarchy of values and aspirations and thereby regulates human behavior (Makselon, 1996). From an existential perspective, death is an integral part of experience, and awareness of mortality can give life meaning (Żemojtel-Piotrowska and Piotrowski, 2009). Death attitudes are multidimensional and encompass, among others, fear, reflection, fascination, belief in life after death, and diverse images of what death entails (Próchniak, 2005). They can also be ambivalent, oscillating between a desire to escape from death and the choice of death as a form of escape (van Beek and Chistopolskaya, 2015a).

Fear of death and uncertainty about what comes after death appear to be natural elements of human thinking (Mavrogiorgou et al., 2020). Terror management theory posits that fear of death is a driving force behind many human behaviors (Żemojtel-Piotrowska and Piotrowski, 2009). Fear of death can be defined as feelings of panic, fear, or anxiety evoked by thoughts about death, the final separation from the world, or what may follow life (Khademi et al., 2021). Research indicates that older adults think about death more frequently than younger people (Timoszyk-Tomczak and Bugajska, 2012). High levels of fear of death in later life may be linked to health problems, chronic illness, mobility limitations, disability, and dependence on others. Retirement and related experiences of loneliness can also contribute to increased fear of death among older adults (Menzies and Menzies, 2020).

The concept of future time perspective (FTP), introduced by Fraisse (1963), refers to how individuals perceive time as a psychological phenomenon. FTP is defined as an acquired and relatively stable set of beliefs about the future (Husman and Shell, 2008; Lens and Rand, 1997; Zimbardo and Boyd, 1999). It is also described as a cognitive-motivational personality characteristic referring to the placement of goals in the near and distant future, which has important motivational and behavioral consequences in the present (Lens et al., 2012). In Zimbardo and Boyd’s (2008) framework, the future constitutes one of several time perspectives that can serve as a frame for organizing and integrating experience. This perspective usually refers to a positive view of the future, but the future can also be conceptualized in negative terms, associated with lack of hope, anxiety, and fear (Carelli et al., 2011). Future anxiety (FA) is defined as a state of uncertainty, tension, worry, and concern arising from beliefs about potential negative events in the future (Zaleski, 1996). Its sources lie in anticipated, ambiguous, and poorly specified events or threats (Zaleski, 2018).

So far, the notion of future time perspective has been described mainly in relation to personal future within this life, encompassing what is important to the self. It covers the time up to one’s death, which can be filled with projects and plans – positive future – and/or concern and apprehension – negative future. However, future time perspective can also be understood more broadly, extending beyond the limits of this life and beyond the self, forming a beyond-the-self perspective. In this context, a future perspective that goes beyond the boundary of death comprises two aspects: a personal dimension – transcendental future – and a beyond-personal dimension – transcendent future (Timoszyk-Tomczak and Bugajska, 2019). Transcendental future refers to goals, values, expectations, and beliefs related to the time after one’s death, extending into infinity (Boyd and Zimbardo, 1997; Zimbardo and Boyd, 2008). Transcendent future goes beyond the framework of personal existence; it concerns the future of others and of the world and is associated with a holistic perception of life and death, also extending into infinity (Timoszyk-Tomczak and Bugajska, 2016, 2019). Framing the future in ways that extend beyond an individual’s physical existence gives this construct an existential and metaphysical dimension. Consequently, thinking about the future in transcendental and transcendent terms is linked to religion and spirituality (Timoszyk-Tomczak and Bugajska, 2019; van Beek and Kairys, 2015b; Ortuño et al., 2011).

Mood is a temporally extended, low-intensity affective state. It arises gradually, usually lacks a clearly identifiable object, is less differentiated, and serves different functional roles than emotions (Keltner et al., 2021). Mood influences how information is processed and may also shape expectations about what is likely to happen (Davidson, 1998). Compared with momentary affect, mood is relatively stable and typically lacks a specific object; it may therefore provide a more enduring emotional context for evaluative processes and the anticipation of the future in late adulthood.

The relationship between mood and time perspective is complex and bidirectional. Time perspective can be seen as a framework that gives coherence and meaning to affective experience, but mood can also modify how different dimensions of time perspective are perceived. Studies by Stolarski et al. (2014) showed that time perspectives are related to current mood, particularly negative and positive past and hedonistic present. In this framework, positive past refers to a warm, nostalgic, and accepting view of one’s past, whereas negative past reflects an adverse, regret-laden, or aversive view of past experiences. Hedonistic present refers to an orientation toward immediate pleasure, novelty, and enjoyment of the present moment, sometimes with limited consideration of future consequences.

Earlier studies indicate that future time perspective may be more strongly linked to mood in demanding situations where task-focused coping is most adaptive, such as highly challenging tasks (Matthews et al., 2006). A meta-analysis confirms robust associations between future time perspective and affective states and mental health: individuals with a strong future perspective report higher happiness and life satisfaction, whereas a restricted and pessimistically perceived future perspective is associated with greater distress, anxiety, and depressive symptoms (Kooij et al., 2018). Moreover, awareness of limited lifetime is associated with lower scores on the extension dimension of future time perspective – people stop planning long-term and focus more on the quality of the here and now. As a result, future time perspective shortens with age and with increased salience of death, and attention shifts from achievement-oriented goals to emotionally meaningful ones (Lang and Carstensen, 2002). Mood is likely an important determinant of future time perspective: positive affect supports viewing the future as full of possibilities, whereas negative mood narrows the temporal horizon (Kooij et al., 2018).

The present study examined how three dimensions of death attitudes – contemplation of death, fear of death, and belief in life after death – together with general mood are related to four dimensions of the future in late adulthood: positive future, negative future, transcendental future, and transcendent future. The study also considered the role of gender, age, and mood in differentiating these future-related dimensions. The theoretical framework for these associations is provided by Socioemotional Selectivity Theory (SST; Carstensen, 2006) and Terror Management Theory (TMT; Greenberg et al., 1986). SST proposes that awareness of limited lifetime changes motivational priorities and increases the importance of emotionally meaningful goals, whereas TMT emphasizes that awareness of mortality and ways of managing death-related anxiety can influence beliefs, meaning-making, and behavior. From these perspectives, both attitudes toward death and general mood may shape future time perspective in late adulthood.

Based on these considerations, we expected general mood to be associated primarily with personal future within this life: more positive general mood with higher positive future, and lower general mood with higher negative future. We further expected fear of death to be associated with higher negative future. Death attitudes, particularly belief in life after death and contemplation of death, were expected to show stronger associations with future beyond one’s own life, including transcendental and transcendent future. We also expected fear of death to be associated with transcendental future, given its focus on the individual’s own existence after death. Age was included as a continuous covariate in the path analysis.

To examine these expectations, we specified an observed-variable path model using composite scores, in which contemplation of death, fear of death, belief in life after death, general mood, and age were modeled as predictors of the four future time perspective dimensions. Preliminary multiple regression analyses were used to examine the pattern of associations and to inform the specification of the path model. In addition, gender, age group, and general mood group were examined as grouping variables in MANOVA to assess mean differences and interaction patterns across the four future dimensions.

## 2 Methods

### 2.1 Participants and procedure

The study was conducted in two stages. In the first phase, 33 participants completed paper-and-pencil questionnaires under the supervision of a psychology student. This phase involved initial recruitment through participants’ local social networks using a snowball sampling procedure. To increase the sample size and broaden the sociodemographic diversity of the sample, a second phase was conducted through the Pollster research center, where 413 participants completed the questionnaires online using a web-based platform. The questionnaire battery included 61 substantive items, in addition to demographic questions. In total, 446 individuals (226 women and 220 men) aged 60 to 94 years participated in the study. All participants resided in Poland. The sample included respondents from different types of place of residence (village: 121, small town: 149, large city: 176) and with different levels of education (primary or vocational: 78, secondary: 208, higher: 160). Regarding religious self-identification, 174 participants (39.2%) identified as believing and practicing, 180 (40.5%) as believing but non-practicing, and 90 (20.3%) as non-believing; two participants did not provide a response.

### 2.2 Measures

To assess the study variables, we used the following instruments: the General Mood Scale (Wojciszke and Baryła, 2005), the short version of the Zimbardo Time Perspective Inventory (ZTPI; Cybis et al., 2012), the Transcendental and Transcendent Future Time Perspective Questionnaire (Timoszyk-Tomczak and Bugajska, 2019), the Dark Future Scale (DFS; Zaleski et al., 2019), and three subscales from the Death Attitude Questionnaire (Próchniak, 2008). For all measures, scores were calculated by summing item responses after reverse-scoring negatively keyed items where applicable.

#### 2.2.1 General mood

General mood (GM) was measured with the General Mood Scale (Wojciszke and Baryła, 2005). The scale consists of 10 items assessing overall positive and negative mood, independent of specific time frames. Participants rate each statement on a five-point scale from 1 (strongly disagree) to 5 (strongly agree). For example, “I feel relaxed and calm”; higher scores indicate a more positive general mood. The scale has shown satisfactory psychometric properties, with internal consistency of *α* = 0.88 (Wojciszke and Baryła, 2005). In the present study, Cronbach’s alpha was *α* = 0.93.

#### 2.2.2 Positive future

Positive future (PF) was assessed with the Future subscale of the short version of the Zimbardo Time Perspective Inventory (ZTPI; Cybis et al., 2012). The short ZTPI comprises 15 items. Respondents rate statements on a five-point Likert scale ranging from 1 (very untrue) to 5 (very true). The inventory measures five time perspectives: two past (positive and negative), two present (hedonistic and fatalistic), and one future. In the present study, only the Future subscale, interpreted as positive future, was used. Example item: “I complete projects on time by making steady progress.”; higher scores indicate a stronger positive future orientation. In previous research, the reliability of this subscale was satisfactory (*α* = 0.71; Cybis et al., 2012). In the current sample, Cronbach’s alpha for the Future subscale was *α* = 0.73.

#### 2.2.3 Transcendental and transcendent future

Transcendental future (TDL) and transcendent future (TDT) were measured using the Transcendental and Transcendent Future Time Perspective Questionnaire (Timoszyk-Tomczak and Bugajska, 2019). The questionnaire consists of 18 items and assesses the extent to which individuals focus on life after death. It comprises two subscales. The transcendental future subscale refers to representations concerning time after one’s own death, extending into infinity and including, for example, expectations of meeting loved ones or salvation, that is, a personal future imagined beyond the end of one’s lifetime. It often co-occurs with belief in some form of life after death, but captures how this belief is integrated into a future-oriented temporal perspective. The transcendent future subscale captures a holistic view of life and death that is less centered on the self and includes, in addition to the personal aspect, generational and metaphysical aspects. Respondents rate each item on a five-point scale from 1 (strongly disagree) to 5 (strongly agree). Example items are “I think about the time after my death” (transcendental future) and “When I am thinking about the future, I am thinking about future generations” (transcendent future); higher scores indicate a stronger orientation toward the respective future dimension. The original questionnaire demonstrated good reliability, with *α* = 0.88 for transcendental future and *α* = 0.81 for transcendent future (Timoszyk-Tomczak and Bugajska, 2019). In the present study, Cronbach’s alpha was *α* = 0.91 for transcendental future and *α* = 0.85 for transcendent future. In the original validation, these two subscales were shown to form related but distinct factors, and in the present sample confirmatory factor analyses likewise supported a two-factor model over a one-factor solution for the 18 items (Δ*χ*^2^(1) = 341.79, *p* < 0.001). Full CFA fit indices and factor loadings for these models are provided in the Supplementary materials.

#### 2.2.4 Negative future

Negative future (NF) was assessed with the Dark Future Scale (DFS; Zaleski et al., 2019). The original instrument developed by Zaleski (1987) is a unidimensional scale comprising 25 items measuring future-related anxiety at both global (e.g., ecological catastrophe) and individual levels (e.g., car accident). The internal consistency of the full scale is *α* = 0.92. In the present study, we used the short, five-item version of the Dark Future Scale (Zaleski et al., 2019). As in the original, participants rate items on a seven-point scale from 0 (definitely no) to 6 (definitely yes). For example, “I am afraid that the problems which trouble me now will continue for a long time”; higher scores indicate greater negative expectations and anxiety concerning the future. The short version has shown good psychometric properties, with Cronbach’s alpha of *α* = 0.88 (Zaleski et al., 2019); in the current study, *α* = 0.86.

#### 2.2.5 Death attitudes

Death attitudes were measured with the Death Attitude Questionnaire (Próchniak, 2008). The instrument consists of 21 items and five subscales: belief in life after death, contemplation of death, fear of death, type of death, and paranormal beliefs about the date of death. Respondents use a seven-point scale from 1 (definitely false) to 7 (definitely true). Example items are “I willingly listen to reflections on death” (contemplation of death), “I am afraid of my own death” (fear of death), and “There is life after death” (belief in life after death); higher scores indicate a higher level of the respective death attitude. In the original study, Cronbach’s alpha for the subscales ranged from 0.75 to 0.86, indicating satisfactory reliability (Próchniak, 2008). In the present study, participants completed three subscales: belief in life after death (BLD), contemplation of death (CD), and fear of death (FD). Their reliability coefficients were *α* = 0.95 for BLD, *α* = 0.83 for CD, and *α* = 0.91 for FD.

### 2.3 Statistical analysis

All analyses were conducted using JASP (Version 0.19.3; JASP Team, 2024). We first computed descriptive statistics and Pearson correlations for all study variables. Because belief in life after death and transcendental future were highly correlated, we additionally tested their measurement structure in brief confirmatory factor analyses, which supported modelling them as two closely related but empirically distinguishable constructs (see Supplementary materials for detailed CFA results). As an initial step, we estimated multiple regression models with each future dimension as an outcome to identify theoretically and empirically supported predictors. These findings were then formalized in an observed-variable path model using composite scores (Model A), in which contemplation of death, belief in life after death, fear of death, general mood, and age predicted the four future time perspective dimensions. Age was included among the predictors because future time perspective, and in particular its transcendental and transcendent dimensions, changes across late adulthood. Model A was evaluated using standard fit indices and compared with more saturated and more parsimonious alternative specifications. Finally, we examined whether the four future dimensions differed between groups defined by gender, age, and general mood. These three characteristics were entered as between-subjects factors in a multivariate analysis of variance with Pillai’s trace, followed by univariate analyses of variance. In the regression and path analyses, age and general mood were treated as continuous variables. For the MANOVA, age was categorized into 60–70 years (*n* = 249) and 71–95 years (*n* = 197) to distinguish younger and older segments of the late-adulthood sample. General mood was standardized, and participants with a standardized score at or below 0 were assigned to the lower-mood group (*n* = 192), whereas those with a score above 0 were assigned to the higher-mood group (*n* = 254). The role of the remaining sociodemographic characteristics was examined in three additional specifications: Model A with gender added as a predictor of all four future dimensions, separate multivariate analyses of variance for place of residence and level of education, and Model A with level of education represented by two dummy variables (primary or vocational education and higher education, with secondary education as the reference category). Detailed output for the regression models, Model A and the alternative specifications, the confirmatory factor analyses, and the additional sociodemographic specifications is reported in the Supplementary materials.

### 2.4 Ethical considerations

The study was conducted in accordance with the ethical standards of the institutional research committee and the 1964 Declaration of Helsinki and its later amendments. Ethical approval was obtained from the Ethics Committee at the Institute of Psychology, University of Szczecin (KB 31/19.09.2024). Participation was voluntary and anonymous, and no identifying information was collected. Participants could discontinue participation at any time without providing a reason and completed the questionnaires at their own pace, with no time limit. Contact details for the research team were provided for questions and concerns about the study. Informed consent was obtained from all participants prior to completing the questionnaires, both in the paper-and-pencil and online versions.

## 3 Results

Analyses combined correlational analyses and path analysis, supplemented by multivariate analyses of variance (MANOVA). Descriptive statistics and Pearson correlations are presented in Table 1, parameter estimates and fit indices for the main path model (Model A) are presented in Table 2, and the MANOVA and follow-up ANOVAs are presented in Table 3.

**Table 1:** Pearson correlations between age, mood, death attitudes, and future time perspective dimensions (*N* = 446).

Variable	1	2	3	4	5	6	7	8	9
1. Age	—								
2. GM	−0.05	—							
3. CD	−0.14**	0.01	—						
4. FD	−0.08	−0.30***	0.00	—					
5. BLD	−0.12*	−0.02	0.46***	0.01	—				
6. TDL	−0.11*	−0.04	0.54***	0.07	0.85***	—			
7. TDT	−0.03	0.07	0.50***	−0.00	0.51***	0.68***	—		
8. NF	−0.03	−0.42***	0.23***	0.34***	0.13**	0.20***	0.23***	—	
9. PF	0.02	0.12**	0.06	−0.08	0.11*	0.13**	0.24***	−0.01	—

GM, general mood; CD, contemplation of death; FD, fear of death; BLD, belief in life after death; TDL, transcendental future; TDT, transcendent future; NF, negative future; PF, positive future. *p < 0.05, **p < 0.01, ***p < 0.001.

**Table 2:** Parameter estimates and model fit for the main path model (Model A; *N* = 446).

Outcome	Predictor	*B*	*SE*	*β*	95% CI for *B*	*p*
Panel A. Structural paths						
Positive future (PF)	General mood (GM)	0.020	0.009	0.103	[0.003, 0.038]	0.024
	Belief in life after death (BLD)	0.028	0.012	0.110	[0.005, 0.052]	0.019
	Age	0.010	0.014	0.035	[−0.017, 0.037]	0.462
Negative future (NF)	General mood (GM)	−0.234	0.026	−0.370	[−0.285, −0.183]	< 0.001
	Contemplation of death (CD)	0.241	0.042	0.228	[0.157, 0.324]	< 0.001
	Fear of death (FD)	0.223	0.041	0.227	[0.144, 0.303]	< 0.001
	Age	−0.001	0.038	−0.001	[−0.075, 0.073]	0.975
Transcendental future (TDL)	Belief in life after death (BLD)	0.790	0.028	0.762	[0.735, 0.845]	< 0.001
	Contemplation of death (CD)	0.244	0.036	0.185	[0.175, 0.314]	< 0.001
	Fear of death (FD)	0.078	0.026	0.063	[0.028, 0.129]	0.002
	Age	0.017	0.028	0.014	[−0.039, 0.072]	0.554
Transcendent future (TDT)	Belief in life after death (BLD)	0.314	0.038	0.350	[0.240, 0.389]	< 0.001
	Contemplation of death (CD)	0.397	0.048	0.347	[0.303, 0.492]	< 0.001
	Age	0.059	0.039	0.058	[−0.018, 0.136]	0.132
Panel B. Model summary						
Explained variance	*R*^2^PF = 0.022; *R*^2^NF = 0.289; *R*^2^TDL = 0.746; *R*^2^TDT = 0.348					
Model fit	*χ*^2^(6) = 8.30, *p* = 0.217; CFI = 0.998; TLI = 0.991; RMSEA = 0.029, 90% CI [0.000, 0.073]; SRMR = 0.014					

*B*, unstandardized regression coefficient; SE, standard error; *β*, standardized regression coefficient; CI, confidence interval. Age was retained as a covariate for all outcomes. Paths not shown were fixed to zero in Model A. Residual covariances among PF, NF, TDL, and TDT were estimated but are not displayed. Bootstrap analyses based on 5,000 resamples are reported in the Supplementary materials.

**Table 3:** MANOVA and follow-up univariate effects for the four future time perspective dimensions.

Dependent variable	Effect	*F*	*df*	*p*	η^2^_p_
Multivariate tests					
	General mood	20.76	4, 435	< 0.001	0.160
	Gender	7.68	4, 435	< 0.001	0.066
	Age group	2.83	4, 435	0.025	0.025
	Mood × gender	0.74	4, 435	0.564	0.007
	Mood × age	0.68	4, 435	0.604	0.006
	Gender × age	1.66	4, 435	0.158	0.015
	Mood × gender × age	2.91	4, 435	0.021	0.026
Univariate tests					
Positive future	General mood	12.47	1, 438	< 0.001	0.028
	Gender	6.03	1, 438	0.014	0.014
	Gender × age	4.84	1, 438	0.028	0.011
Negative future	General mood	60.72	1, 438	< 0.001	0.122
	Gender	13.61	1, 438	< 0.001	0.030
	Mood × gender × age	10.40	1, 438	0.001	0.023
Transcendental future	Gender	18.19	1, 438	< 0.001	0.040
Transcendent future	Gender	11.55	1, 438	< 0.001	0.026

*N* = 446. Multivariate tests are based on Pillai’s trace; for effects with one numerator degree of freedom, Pillai’s trace is numerically equivalent to partial eta squared. Univariate tests are based on Type III sums of squares, and η^2^p was computed as SSeffect/(SSeffect + SSresidual). Mood = general mood, standardized and dichotomized at *z* = 0 (lower mood: *n* = 192; higher mood: *n* = 254); age group = 60–70 years (*n* = 249) versus 71–95 years (*n* = 197). Only univariate effects significant at *p* < 0.05 are shown. Complete univariate results, including non-significant effects, tests of simple effects for the two significant interactions, and the corresponding cell means are reported in the Supplementary materials, where the two interactions are also displayed graphically.

Overall, the pattern of results suggests that lower general mood is consistently associated with a more negative view of the personal future within this life (higher negative future) and with higher fear of death, whereas belief in life after death and contemplation of death are particularly strongly related to future orientations beyond one’s own life (transcendental future, and transcendent future). In addition, women reported higher levels of transcendental and transcendent future than men. The effect of gender on positive future differed by age group, whereas the association between general mood and negative future differed across combinations of gender and age.

### 3.1 Correlations

Pearson correlations between the study variables are shown in Table 1. Age was negatively correlated with contemplation of death, belief in life after death, and transcendental future, with coefficients ranging from −0.11 to −0.14. General mood was negatively related to negative future and fear of death: lower general mood was associated with higher negative future and greater fear of death. General mood also showed a small positive correlation with positive future, such that higher general mood was associated with slightly higher positive future. Among the death-attitude variables, contemplation of death was strongly and positively associated with belief in life after death.

Contemplation of death was positively correlated with transcendental future, transcendent future, and negative future: higher levels of contemplation of death were related to higher transcendental and transcendent future and to a more negative view of the future. Fear of death was positively related to negative future, indicating that individuals with greater fear of death reported a more negative view of the future. Belief in life after death was significantly associated with all four future dimensions, with particularly strong correlations with transcendental and transcendent future and very weak correlations with negative and positive future. Transcendental and transcendent future were strongly intercorrelated and positively related to both negative and positive future. Negative and positive future were not significantly correlated with each other.

### 3.2 Path model (Model A)

Guided by the preliminary regression analyses, we specified the observed-variable path model using composite scores (Model A) in which contemplation of death, belief in life after death, fear of death, general mood and age predicted the four future time perspective dimensions: positive future, negative future, transcendental future, and transcendent future. Residual covariances were estimated among positive future, negative future, transcendental future, and transcendent future to account for shared variance not explained by the predictors. Model A showed very good fit to the data (*χ*^2^(6) = 8.30, *p* = 0.217, CFI = 0.998, TLI = 0.991, RMSEA = 0.029, 90% CI [0.000, 0.073], SRMR = 0.014; see Table 2).

In this model, negative future was primarily predicted by lower general mood and higher fear of death and contemplation of death, with general mood showing the strongest association (*B* = −0.234, SE = 0.026, *z* = −8.998, *p* < 0.001). Contemplation of death and fear of death were both positively related to negative future (contemplation of death: *B* = 0.241, SE = 0.042, *z* = 5.672, *p* < 0.001; fear of death: *B* = 0.223, SE = 0.041, *z* = 5.500, *p* < 0.001), whereas age did not contribute uniquely (*B* = −0.001, *p* = 0.975). Positive future showed small positive associations with general mood and belief in life after death (general mood: *B* = 0.020, SE = 0.009, *z* = 2.256, *p* = 0.024; belief in life after death: *B* = 0.028, SE = 0.012, *z* = 2.338, *p* = 0.019), whereas contemplation of death, fear of death, and age were not significant predictors.

Transcendental future was strongly predicted by belief in life after death, with additional positive contributions from contemplation of death and fear of death and no independent effect of age (belief in life after death: *B* = 0.790, SE = 0.028, *z* = 28.346, *p* < 0.001; contemplation of death: *B* = 0.244, SE = 0.036, *z* = 6.862, *p* < 0.001; fear of death: *B* = 0.078, SE = 0.026, *z* = 3.044, *p* = 0.002; age: *B* = 0.017, *p* = 0.554). Transcendent future was best explained by contemplation of death and belief in life after death (contemplation of death: *B* = 0.397, SE = 0.048, *z* = 8.245, *p* < 0.001; belief in life after death: *B* = 0.314, SE = 0.038, *z* = 8.303, *p* < 0.001), again without a significant age effect (*B* = 0.059, *p* = 0.132). The proportion of variance explained by Model A was *R*^2^ = 0.289 for negative future, *R*^2^ = 0.022 for positive future, *R*^2^ = 0.746 for transcendental future, and *R*^2^ = 0.348 for transcendent future, indicating that the model accounted for a substantial share of variance in negative, transcendental, and transcendent future, but only a small proportion of variance in positive future.

Separate multiple regression models were estimated as an initial analysis to inform the specification of Model A; their full results are reported in the Supplementary materials. Model A was further evaluated against several alternative path models: a saturated reference model (Model B), in which all five predictors were allowed to predict all four future dimensions; a composite “afterlife” model (Model C), which collapsed belief in life after death and transcendental future into a single index; and a two-domain future model (Model D), which grouped positive and negative future into a within-life future factor and transcendental and transcendent future into a beyond-life future factor. Although the saturated model closely reproduced the covariance structure, its information criteria were slightly higher than those of Model A, and the composite and two-domain models showed clearly poorer fit and less stable parameter estimates. Thus, Model A emerged as the most parsimonious and empirically well-supported representation of the relations between death attitudes, general mood, age, and the four future time perspective dimensions. As a sensitivity analysis, Model A was re-estimated with gender included as an additional predictor of all four future dimensions. The focal path estimates and their statistical significance were substantively unchanged; detailed results are reported in the Supplementary materials. In a further sensitivity analysis, Model A was re-estimated with level of education included as a predictor of all four future dimensions. The associations of general mood and belief in life after death with positive future remained positive and statistically significant, and the remaining path estimates were substantively unchanged. Relative to secondary education, higher education was associated with higher positive future, and explained variance in positive future increased from *R*^2^ = 0.022 to *R*^2^ = 0.041. Detailed results are reported in the Supplementary materials. To assess parameter uncertainty under deviations from multivariate normality, we also estimated bootstrap confidence intervals for the Model A paths; these intervals closely matched the asymptotic confidence intervals reported in Table 2, indicating stable estimates.

### 3.3 MANOVA and group differences

Next, we examined whether future-related variables differed across groups defined by gender, age, and general mood. First, we examined the assumptions of MANOVA. The Shapiro–Wilk test indicated deviations from normality (W = 0.987, *p* < 0.001), and Box’s M test indicated a marginal departure from homogeneity of the covariance matrices, *χ*^2^(70) = 91.86, *p* = 0.041. Cell sizes were moderately large and approximately balanced (*n* = 44–74). Correlations among the dependent variables were moderate (*r* ≤ 0.68), below the commonly accepted threshold for multicollinearity (*r* > 0.80; PQStat Software, 2014). Visual inspection of boxplots suggested no multivariate outliers. Given the robustness of Pillai’s trace to deviations from normality, unequal group sizes, and minor violations of homogeneity of covariance matrices (Olson, 1976; Wilks, 1932; IBM Corp, 2023), we used Pillai’s trace as the multivariate test statistic (Tabachnick and Fidell, 2019; Pietrzak, 2020).

In the MANOVA, the four future dimensions—positive future, negative future, transcendental future, and transcendent future—were analyzed jointly as dependent variables. Gender, age group, and general mood group were entered as between-subjects factors (see Table 3).

The multivariate tests showed significant effects of general mood, gender, and age group, as well as a significant three-way interaction between general mood, gender, and age group. General mood had the strongest multivariate effect (Pillai’s V = 0.160, partial η^2^ = 0.160), followed by gender (Pillai’s V = 0.066, partial η^2^ = 0.066) and age group (Pillai’s V = 0.025, partial η^2^ = 0.025).

Follow-up univariate ANOVAs revealed several specific effects. Gender significantly differentiated transcendental future (*F*(1, 438) = 18.19, *p* < 0.001, partial η^2^ = 0.040) and transcendent future (*F*(1, 438) = 11.55, *p* < 0.001, partial η^2^ = 0.026), with women scoring higher on both beyond-life future dimensions than men. The gender effects for positive and negative future were qualified by significant interactions reported below. General mood significantly differentiated negative future (*F*(1, 438) = 60.72, *p* < 0.001, partial η^2^ = 0.122), such that lower general mood was associated with higher negative future, and also differentiated positive future (*F*(1, 438) = 12.47, *p* < 0.001, partial η^2^ = 0.028), with higher general mood associated with higher positive future.

Furthermore, the effect of gender on positive future depended on age group (gender × age interaction: *F*(1, 438) = 4.84, *p* = 0.028, partial η^2^ = 0.011). In the 60–70 age group, women reported higher positive future than men, whereas in the 71–95 age group no meaningful gender difference was observed (M = 11.79 for men and M = 11.78 for women). A significant three-way interaction between general mood, gender, and age group emerged for negative future (*F*(1, 438) = 10.40, *p* = 0.001, partial η^2^ = 0.023), indicating that the association between general mood and negative future differed across combinations of gender and age. Simple-effects tests showed that the association between lower general mood and higher negative future was especially strong in younger women and older men, whereas it was weaker in older women and younger men.

Finally, the four future dimensions were compared across levels of place of residence and education. Place of residence showed no multivariate effect (Pillai’s V = 0.028, *F*(8, 882) = 1.57, *p* = 0.131, partial η^2^ = 0.014). Level of education showed a small multivariate effect (Pillai’s V = 0.036, *F*(8, 882) = 2.03, *p* = 0.040, partial η^2^ = 0.018), which was confined to positive future (*F*(2, 443) = 4.58, *p* = 0.011, partial η^2^ = 0.020); participants with higher education reported slightly higher positive future than those with secondary education (ΔM = 0.52, *p* = 0.015). Neither variable differentiated the remaining three dimensions.

## 4 Discussion

The present study examined how three dimensions of death attitudes – contemplation of death, fear of death, and belief in life after death – together with general mood are related to four dimensions of the future in late adulthood: positive future, negative future, transcendental future, and transcendent future, taking into account the role of gender and age in differentiating the four future dimensions. The findings partly confirmed the expected associations between these variables and highlighted both the specific role of mood and the distinct patterns linking death attitudes with different dimensions of future orientation.

The correlational analyses showed that lower general mood was associated with a more negative view of the future and with higher fear of death. In the context of death attitudes, higher contemplation of death was related to higher transcendental future and transcendent future as well as to a more negative future but was not related to a more positive future. Higher fear of death was also associated with a more negative future. Belief in life after death was significantly related to all four dimensions of future orientation, with particularly strong correlations with transcendental future and transcendent future. Small age related associations were observed for contemplation of death, belief in life after death, and transcendental future. In addition, men and women differed significantly on all future scales.

The regression models allowed us to identify predictors of the different aspects of future orientation. On this basis, we specified a coherent path model in which the predictor variables general mood, belief in life after death, contemplation of death, fear of death, and age jointly predicted the four future dimensions. The path analyses showed different patterns for positive and negative personal future within this life. Positive future was related to higher general mood and belief in life after death and was not independently explained by fear or contemplation of death, whereas negative future was associated with lower general mood and higher fear of death and contemplation of death. The associations of positive future with general mood and belief in life after death should be interpreted cautiously, given the limited explanatory value of the model for positive future. Beyond-life future was primarily linked to death attitudes rather than to mood: both transcendental and transcendent were predicted by belief in life after death and contemplation of death, and transcendental future was additionally explained by fear of death. Age did not uniquely predict any of the four future dimensions once mood and death attitudes were taken into account. This pattern may support the notion that an open, future-oriented outlook reduces depressive tendencies (Coudin and Lima, 2011). The observed relationships are consistent with previous findings showing that a limited personal time horizon is linked to the interplay between positive mood and a sense of meaning in life (Hicks et al., 2012).

Transcendental future refers to representations of “what will happen to me after death”, that is, to time after one’s own death that still involves the self. Transcendent future also concerns time after death but extends beyond the self and focuses on what will happen to others and to the world, and on the broader meaning of existence (Timoszyk-Tomczak and Bugajska, 2019). The present results indicate that both death attitudes and mood are related to future orientation. General mood is primarily associated with personal future within this life (positive and negative), whereas thinking about future beyond this life from a beyond-the-self perspective (transcendent future) appears to be largely independent of current mood. Death attitudes are relevant for both future within this life (positive and negative) and future beyond this life (transcendental and transcendent), and for both personal future (positive, negative, transcendental) and future extending beyond the personal context (transcendent). Overall, the findings suggest that mood organizes the tone of personal, within-life future, while fear of death predicts negative future within this life and transcendental future beyond this life.

Several mechanisms may underlie these patterns. One possibility is that current mood determines the valence of within-life future time perspective, whereas death attitudes shape the beyond-life future perspective. More specifically, the valence of personal future within this life seems to rest on an affective–worldview dynamic. Positive future is shaped by positive mood and belief in life after death, which is consistent with the broaden-and-build theory (Fredrickson, 2001), whereby positive affect broadens cognitive resources and facilitates planning, while belief in postmortem continuity provides a basis for meaning (Greenberg et al., 1986). In turn, negative future is explained by lower mood, higher fear of death, and higher contemplation of death. Under conditions of reduced mood and heightened existential anxiety, beliefs about continuity after death may lose their soothing function and become a source of additional concerns (e.g., about the uncertainty of one’s postmortem fate), thereby amplifying pessimistic views of the future.

The beyond-life dimensions differentiate according to the level of confrontation with fear of death. Transcendent future, understood as a more impersonal stream of existence, appears to require mainly a cognitive framework in the form of belief in life after death and reflective contemplation of death. Transcendental future, which focuses on personal “continuation” of life after death, additionally activates fear of death. In light of Terror Management Theory (Greenberg et al., 1986), this suggests that the vision of a personal postmortem future may serve a direct buffering function—helping to manage fear of annihilation and protect the self. Issues that directly concern the self tend to be more engaging and are more likely to activate emotional processes (Frijda, 1996), which may further explain the association between personal future and both mood and fear of death. What is more distant in time, such as transcendent future, tends to elicit more abstract, decontextualized thinking (Liberman and Trope, 1998; Trope and Liberman, 2003). Psychological distance attenuates affective responses (Frijda, 1986), which may limit emotional reactions to transcendent categories. The study focused on individuals in late adulthood, a life period in which people are more aware of time and more conscious that their lifetime will end (Carstensen, 2006). Death may therefore be experienced as more real, which can increase fear and stress with regard to personal future. At the same time, we cannot rule out the reverse direction of influence – that lowered mood may increase the frequency of thoughts about difficult or threatening topics (Bower, 1981). Studies by Charles and Carstensen (2010) also suggest that when time is perceived as open, knowledge related goals gain importance, whereas when time is experienced as limited, emotionally meaningful goals become paramount.

The findings highlight several important regularities. First, perceptions of the future in old age are not fixed and may vary in relation to general mood. This aligns with Socioemotional Selectivity Theory, which emphasizes age related shortening of the temporal horizon alongside prioritization of the regulation of emotions experienced in the here and now (Carstensen, 2021). In this context, a strongly negative view of the future in the presence of low mood may indicate that older adults with reduced emotional well being view the upcoming time primarily through the lens of losses, a pattern sometimes described as geriatric pessimism. It should also be emphasized that the observed associations were generally modest and do not rule out reverse or reciprocal relationships.

A second regularity concerns higher transcendent and transcendental future in women. On average, women in late adulthood tend to shift their perspective from a more materialistic rational stance toward a more symbolic and spiritual one. This is consistent with Lars Tornstam’s concept of gerotranscendence (Tornstam, 2011, 2020) and with other studies showing that older women are more inclined than men to seek and develop the spiritual dimension in later life (Braam et al., 2006).

An interesting, albeit small, finding was the interaction between gender and age group for positive future. Women aged 60–70 years reported higher positive future than men in the same age group, whereas no meaningful gender difference was observed among participants aged 71–95 years. Thus, the interaction reflected a convergence of gender differences in positive future across age groups rather than an advantage of older men. A similar emphasis on context is suggested by the finding that level of education was related only to positive future, the dimension least explained by death attitudes and mood, indicating that how older adults envisage a positive personal future may depend more on social and material resources than on their relation to death. Future research should examine whether the convergence in positive future is related to changing social roles, health, social support, or other contextual factors in later adulthood.

The modest but statistically significant three-way interaction between general mood, gender, and age group for negative future suggests that negative future is not a simple function of age or mood alone. The association between lower general mood and higher negative future was especially strong among women aged 60–70 years and men aged 71–95 years, whereas it was weaker among older women and younger men. Psychological resilience may play an important role in how older adults manage negative affect and future-related concerns (Windle et al., 2010). This pattern should nevertheless be interpreted cautiously, given the small effect size. Future research should examine whether resilience, social support, health, and life circumstances help explain differences in the association between mood and negative future across gender and age groups in late adulthood.

Despite the statistically significant findings, the present study has several limitations. Its cross-sectional and correlational design precludes causal inferences, and the self-report measures may have introduced response biases. The dominant role of general mood in explaining variance in the dependent variables points to the need for longitudinal designs in future research, which would make it possible to distinguish stable personality dispositions from situational states. Moreover, the intriguing interaction between gender and age in positive future suggests that future studies should incorporate additional sociodemographic factors, such as marital and family status or functional health. Future research could focus on identifying the mechanisms behind the convergence of positive future in men and women after the age of 70, that is, why the initial advantage observed among women aged 60–70 disappears in the older age group, which could provide a basis for targeted psychogerontological interventions.

Overall, the study underscores that how older adults imagine both their future within this life and their beyond-life future is closely intertwined with their mood and with the ways they relate to death, and may therefore be an important focus for assessment and intervention in late adulthood.

## Data Availability

The raw data supporting the conclusions of this article will be made available by the authors, without undue reservation.
