# Supplementary material for: Beyond the here and now: mood, death attitudes, and diverse future time perspective in late adulthood
Source: Front Psychol. 2026 Sep 23;17:1921975. doi: 10.3389/fpsyg.2026.1921975 (PMC13642270; doi:10.3389/fpsyg.2026.1921975)
Supplement: Supplementary file 1 [file Supplementary_file_1.DOCX]

**Supplementary Material**

Beyond the Here and Now: Belief in Life After Death, Death Contemplation, and Fear of Death as Predictors of Future Time Perspective in Older Adults

All analyses were conducted in JASP on the full sample of N = 446 community-dwelling older adults (aged 60–94 years). Estimation was by maximum likelihood. Variable abbreviations: GM = general mood; CD = contemplation of death; FD = fear of death; BLD = belief in life after death; PF = positive future; NF = negative future; TDL = transcendental future (life-related); TDT = transcendent future (transcendence-related).

# **Contents**

**Table S1.** Multiple regression analyses predicting the four future time perspective dimensions

**Table S2.** Unstandardized and standardized parameter estimates for Model A

**Table S3.** Residual covariances among the four outcome variables in Model A

**Table S4.** Goodness-of-fit statistics for Model A and the three alternative models

**Table S5.** Confirmatory factor analyses supporting the measurement structure

**Table S6.** Gender-adjusted sensitivity specification of Model A

**Table S7.** Multivariate and univariate analyses of variance for place of residence and level of education

**Table S8.** Descriptive statistics and post hoc comparisons by level of education

**Table S9.** Education-adjusted sensitivity specification of Model A

**Table S10.** Multivariate analysis of variance (Pillai’s trace)

**Table S11.** Univariate analyses of variance with effect sizes

**Table S12.** Cell means for the significant interaction effects

**Table S13.** Simple effects analyses for the two significant interaction effects

**Figure S1.** Path diagram for Model A with standardized estimates

**Figure S2.** Gender × age interaction for positive future

**Figure S3.** Gender × age × general mood interaction for negative future

**Table S1**

*Multiple Regression Analyses Predicting the Four Future Time Perspective Dimensions (All Predictors Entered Simultaneously)*

| Predictor | B | SE | β | t | p | 95% CI LL | 95% CI UL | VIF |
| --- | --- | --- | --- | --- | --- | --- | --- | --- |
| **Positive future (PF). R² = .029, adjusted R² = .018, F(5, 440) = 2.658, p = .022** | | | | | | | | |
| Age | 0.010 | 0.014 | .034 | 0.701 | .483 | −0.018 | 0.037 | 1.035 |
| GM | 0.022 | 0.010 | .113 | 2.289 | .023 | 0.003 | 0.041 | 1.105 |
| CD | 0.006 | 0.017 | .018 | 0.334 | .739 | −0.029 | 0.040 | 1.282 |
| FD | −0.013 | 0.015 | −.041 | −0.825 | .410 | −0.043 | 0.017 | 1.109 |
| BLD | 0.027 | 0.014 | .103 | 1.940 | .053 | −0.0003 | 0.053 | 1.277 |
| **Negative future (NF). R² = .281, adjusted R² = .273, F(5, 440) = 34.377, p < .001** | | | | | | | | |
| Age | 0.001 | 0.038 | .002 | 0.038 | .969 | −0.073 | 0.076 | 1.035 |
| GM | −0.221 | 0.027 | −.352 | −8.294 | < .001 | −0.274 | −0.169 | 1.105 |
| CD | 0.229 | 0.048 | .219 | 4.790 | < .001 | 0.135 | 0.323 | 1.282 |
| FD | 0.230 | 0.042 | .234 | 5.504 | < .001 | 0.148 | 0.311 | 1.109 |
| BLD | 0.020 | 0.037 | .025 | 0.537 | .592 | −0.054 | 0.094 | 1.277 |
| **Transcendental future (TDL). R² = .746, adjusted R² = .743, F(5, 440) = 258.595, p < .001** | | | | | | | | |
| Age | 0.016 | 0.029 | .014 | 0.575 | .566 | −0.040 | 0.073 | 1.035 |
| GM | −0.005 | 0.020 | −.006 | −0.246 | .806 | −0.044 | 0.034 | 1.105 |
| CD | 0.245 | 0.036 | .185 | 6.802 | < .001 | 0.174 | 0.316 | 1.282 |
| FD | 0.076 | 0.031 | .062 | 2.435 | .015 | 0.015 | 0.138 | 1.109 |
| BLD | 0.790 | 0.028 | .762 | 28.054 | < .001 | 0.735 | 0.846 | 1.277 |
| **Transcendent future (TDT). R² = .357, adjusted R² = .350, F(5, 440) = 48.886, p < .001** | | | | | | | | |
| Age | 0.066 | 0.040 | .065 | 1.664 | .097 | −0.012 | 0.144 | 1.035 |
| GM | 0.059 | 0.028 | .085 | 2.126 | .034 | 0.004 | 0.113 | 1.105 |
| CD | 0.397 | 0.050 | .346 | 7.991 | < .001 | 0.300 | 0.495 | 1.282 |
| FD | 0.027 | 0.043 | .025 | 0.633 | .527 | −0.058 | 0.113 | 1.109 |
| BLD | 0.320 | 0.039 | .355 | 8.219 | < .001 | 0.243 | 0.396 | 1.277 |

*Note.* N = 446. All five predictors were entered simultaneously in a single block (forced entry); no automated variable-selection procedure was used. B = unstandardized coefficient; β = standardized coefficient; CI = confidence interval for B; LL = lower limit; UL = upper limit; VIF = variance inflation factor. Tolerance values ranged from .780 to .966 and all VIFs were below 1.29, indicating no multicollinearity. Durbin–Watson statistics ranged from 1.93 to 2.09.

Two coefficients in Table S1 deserve comment because they differ marginally from the corresponding paths in Model A. First, BLD → PF is borderline in the regression (β = .103, p = .053) but significant in Model A (β = .110, p = .019); the difference reflects the fact that Model A estimates residual covariances among the four outcomes, which reduces the standard errors of the structural paths. Second, GM → TDT reaches conventional significance in the regression (β = .085, p = .034) but was not retained in Model A. This path accounts for approximately 0.7% of unique variance in TDT, and its omission — together with the omission of BLD → NF, FD → TDT, and all four age paths — is jointly tested by the chi-square statistic of the over-identified Model A, which was non-significant, χ²(6) = 8.30, p = .217. Model A therefore provides an adequate account of the covariance structure without these paths, and they were excluded on grounds of parsimony.

**Table S2**

*Unstandardized and Standardized Parameter Estimates for Model A*

| Path | B | SE | z | p | β | Asymptotic 95% CI | | Bootstrap 95% CI | |
| --- | --- | --- | --- | --- | --- | --- | --- | --- | --- |
|  |  |  |  |  |  | *LL* | *UL* | *LL* | *UL* |
| BLD → TDL | 0.790 | 0.028 | 28.346 | < .001 | .762 | 0.735 | 0.845 | 0.735 | 0.841 |
| CD → TDL | 0.244 | 0.036 | 6.862 | < .001 | .185 | 0.175 | 0.314 | 0.172 | 0.317 |
| FD → TDL | 0.078 | 0.026 | 3.044 | .002 | .063 | 0.028 | 0.129 | 0.021 | 0.135 |
| Age → TDL | 0.017 | 0.028 | 0.592 | .554 | .014 | −0.039 | 0.072 | −0.041 | 0.072 |
| BLD → TDT | 0.314 | 0.038 | 8.303 | < .001 | .350 | 0.240 | 0.389 | 0.234 | 0.395 |
| CD → TDT | 0.397 | 0.048 | 8.245 | < .001 | .347 | 0.303 | 0.492 | 0.302 | 0.492 |
| Age → TDT | 0.059 | 0.039 | 1.507 | .132 | .058 | −0.018 | 0.136 | −0.020 | 0.134 |
| GM → NF | −0.234 | 0.026 | −8.998 | < .001 | −.370 | −0.285 | −0.183 | −0.290 | −0.178 |
| CD → NF | 0.241 | 0.042 | 5.672 | < .001 | .228 | 0.157 | 0.324 | 0.147 | 0.335 |
| FD → NF | 0.223 | 0.041 | 5.500 | < .001 | .227 | 0.144 | 0.303 | 0.132 | 0.312 |
| Age → NF | −0.001 | 0.038 | −0.031 | .975 | −.001 | −0.075 | 0.073 | −0.077 | 0.070 |
| GM → PF | 0.020 | 0.009 | 2.256 | .024 | .103 | 0.003 | 0.038 | 0.002 | 0.040 |
| BLD → PF | 0.028 | 0.012 | 2.338 | .019 | .110 | 0.005 | 0.052 | 0.002 | 0.054 |
| Age → PF | 0.010 | 0.014 | 0.736 | .462 | .035 | −0.017 | 0.037 | −0.017 | 0.037 |
| **Explained variance: R² = .022 (PF), .289 (NF), .746 (TDL), .348 (TDT)** | | | | | | | | | |

*Note.* N = 446. B = unstandardized estimate; β = fully standardized estimate. Asymptotic confidence intervals are based on the delta method; bootstrap confidence intervals are percentile intervals from 1,000 resamples. Model fit: χ²(6) = 8.301, p = .217, CFI = .998, TLI = .991, RMSEA = .029, 90% CI [.000, .073], SRMR = .014, AIC = 9,459.51, BIC = 9,574.32, 28 free parameters, log-likelihood = −4,701.76. Mardia’s multivariate skewness was 4.885, χ²(165) = 363.15, p < .001, and multivariate kurtosis was 108.049, z = 6.791, p < .001; the close correspondence between the asymptotic and bootstrap intervals indicates that the departure from multivariate normality did not materially affect inference.

**Table S3**

*Residual Covariances Among the Four Outcome Variables in Model A*

| Covariance | Estimate | SE | z | p | Standardized | 95% CI | |  |
| --- | --- | --- | --- | --- | --- | --- | --- | --- |
| PF – NF | 0.316 | 0.405 | 0.779 | .436 | .037 | [−0.479, 1.110] | |  |
| PF – TDL | 0.572 | 0.306 | 1.871 | .061 | .089 | [−0.027, 1.171] | |  |
| PF – TDT | 1.981 | 0.433 | 4.578 | < .001 | .222 | [1.133, 2.829] | |  |
| NF – TDL | 1.373 | 0.835 | 1.644 | .100 | .078 | [−0.264, 3.009] | |  |
| NF – TDT | 4.785 | 1.177 | 4.064 | < .001 | .196 | [2.477, 7.093] | |  |
| TDL – TDT | 9.161 | 0.971 | 9.437 | < .001 | .500 | [7.258, 11.063] | |  |

*Note.* N = 446. All six residual covariances among the outcome variables were freely estimated in Model A. Confidence intervals are asymptotic.

**Table S4**

*Goodness-of-Fit Statistics for Model A and the Three Alternative Models*

| Model | χ² | df | p | CFI | TLI | RMSEA | 90% CI | SRMR | AIC | BIC |
| --- | --- | --- | --- | --- | --- | --- | --- | --- | --- | --- |
| A (final) | 8.301 | 6 | .217 | .998 | .991 | .029 | [.000, .073] | .014 | 9,459.51 | 9,574.32 |
| B (saturated) | 0.000 | 0 | — | 1.000 | 1.000 | .000 | — | < .001 | 9,463.21 | 9,602.62 |
| C (composite) | 48.662 | 8 | < .001 | .978 | .916 | .107 | [.079, .137] | .034 | 11,775.62 | 11,906.83 |
| D (two domains) | 94.361 | 11 | < .001 | .925 | .823 | .130 | [.107, .155] | .058 | 9,535.57 | 9,629.88 |

*Note.* N = 446. Model A is the final model reported in the main text. Model B is a saturated reference model in which all five predictors were allowed to predict all four outcomes; because it is just-identified it fits perfectly by construction, yet its AIC and BIC are higher than those of Model A, indicating that the additional paths do not improve parsimony-adjusted fit. Model C replaces BLD and TDL with a single composite “afterlife” index; Model D groups PF and NF into a within-life latent factor and TDL and TDT into a beyond-life latent factor. Because Model C is estimated on a different set of observed variables, its AIC and BIC are not directly comparable with those of the other models; the conclusion that it fits less well rests on CFI, TLI, RMSEA, and SRMR. Nested comparisons against Model A: Model C, Δχ²(2) = 40.361, p < .001; Model D, Δχ²(3) = 42.470, p < .001. Numbers of free parameters were 28 (A), 34 (B), 32 (C), and 23 (D).

An additional sensitivity model adding the path FD → TDT to Model A was also examined and did not improve fit, Δχ²(1) = 0.040, p = .841; AIC = 9,461.47 versus 9,459.51 for Model A. Four further exploratory specifications (a model treating GM as an exogenous common cause, a mediation model with CD as a central hub, and two models reversing the direction of the BLD–TDL association) were rejected on the basis of clearly inadequate fit (CFI ranging from .419 to .999 with RMSEA ranging from .056 to .341 and AIC ranging from 15,547.91 to 24,068.57) and are not reported in detail.

**Table S5**

*Confirmatory Factor Analyses Supporting the Measurement Structure*

| Model | χ² | df | CFI | TLI | RMSEA | SRMR | Δχ² |
| --- | --- | --- | --- | --- | --- | --- | --- |
| **Transcendental Future Time Perspective Scale (18 items)** | | | | | | | |
| One-factor | 870.353 | 135 | — | — | — | — |  |
| Two-factor (TDL, TDT) | 528.559 | 134 | — | — | — | — | 341.794*** |
| **Discriminant validity of BLD versus TDL (12 items)** | | | | | | | |
| One-factor | 329.286 | 54 | .940 | .926 | .107 | .040 |  |
| Two-factor (BLD, TDL) | 163.926 | 53 | .976 | .970 | .069 | .028 | 165.360*** |

*Note.* N = 446. ML estimation. Δχ² is the chi-square difference relative to the preceding one-factor model; both two-factor solutions fit significantly better. Additional fit indices were not requested for the Transcendental Future Time Perspective Scale models. For the two-factor TDL/TDT solution, coefficient ω was .917 (TDL) and .853 (TDT) and Cronbach’s α was .912 and .848, respectively; average variance extracted was .586 and .369 and the heterotrait–monotrait ratio was .775. For the two-factor BLD/TDL solution, ω was .947 and .907 and α was .947 and .912, with average variance extracted of .817 and .583 and a heterotrait–monotrait ratio of .899. Although the observed correlation between BLD and TDL was high (r = .846), the two-factor model was clearly superior, supporting their treatment as distinct constructs.

*** p < .001.

**Table S6**

*Gender-Adjusted Sensitivity Specification of Model A Predicting the Four Future Time Perspective Dimensions*

| Predictor | *B* | SE | *z* | *p* | *β* | 95% CI for B |
| --- | --- | --- | --- | --- | --- | --- |
| *Positive future (PF)* | | | | | | |
| GM | 0.022 | 0.009 | 2.479 | .013 | .114 | [0.005, 0.040] |
| BLD | 0.024 | 0.012 | 1.968 | .049 | .094 | [0.0001, 0.048] |
| Age | 0.012 | 0.014 | 0.905 | .365 | .043 | [−0.014, 0.039] |
| Gender | −0.377 | 0.171 | −2.209 | .027 | −.105 | [−0.711, −0.042] |
| *Negative future (NF)* | | | | | | |
| GM | −0.231 | 0.026 | −8.887 | < .001 | −.365 | [−0.282, −0.180] |
| CD | 0.233 | 0.042 | 5.486 | < .001 | .221 | [0.150, 0.316] |
| FD | 0.214 | 0.041 | 5.225 | < .001 | .217 | [0.134, 0.294] |
| Age | 0.004 | 0.038 | 0.095 | .924 | .004 | [−0.070, 0.078] |
| Gender | −0.855 | 0.468 | −1.826 | .068 | −.074 | [−1.773, 0.063] |
| *Transcendental future (TDL)* | | | | | | |
| BLD | 0.784 | 0.028 | 27.940 | < .001 | .756 | [0.729, 0.839] |
| CD | 0.243 | 0.036 | 6.831 | < .001 | .183 | [0.173, 0.312] |
| FD | 0.074 | 0.026 | 2.843 | .004 | .060 | [0.023, 0.125] |
| Age | 0.020 | 0.028 | 0.689 | .491 | .017 | [−0.036, 0.075] |
| Gender | −0.548 | 0.354 | −1.548 | .122 | −.038 | [−1.241, 0.146] |
| *Transcendent future (TDT)* | | | | | | |
| BLD | 0.307 | 0.038 | 8.049 | < .001 | .342 | [0.232, 0.382] |
| CD | 0.395 | 0.048 | 8.201 | < .001 | .344 | [0.301, 0.489] |
| Age | 0.064 | 0.039 | 1.625 | .104 | .063 | [−0.013, 0.141] |
| Gender | −0.806 | 0.485 | −1.663 | .096 | −.064 | [−1.757, 0.144] |

*Note. N* = 446. Gender was coded 0 = women and 1 = men. *B* = unstandardized regression coefficient; *SE* = standard error; *β* = fully standardized estimate; CI = confidence interval for *B*. This sensitivity specification retained the residual covariances among PF, NF, TDL, and TDT estimated in Model A. Model fit: χ²(6) = 9.202, *p* = .163, CFI = .997, TLI = .986, RMSEA = .035, 90% CI [.000, .076], SRMR = .014, AIC = 9,457.92, BIC = 9,589.13, 32 free parameters, log-likelihood = −4,696.96. Explained variance: *R²* = .033 (PF), .296 (NF), .747 (TDL), .353 (TDT). The main path estimates were substantively unchanged relative to Model A. The only significant gender path was that predicting positive future, which converges with the univariate effect of gender on this dimension reported in Table S11.

**Table S7**

*Multivariate and Univariate Analyses of Variance for Place of Residence and Level of Education*

| Effect | Pillai’s *V* | *F* | df | *p* | *η*²*_p_* |
| --- | --- | --- | --- | --- | --- |
| *Place of residence* | | | | | |
| Multivariate test | .028 | 1.566 | 8, 882 | .131 | .014 |
| Positive future (PF) |  | 2.657 | 2, 443 | .071 | .012 |
| Negative future (NF) |  | 1.875 | 2, 443 | .155 | .008 |
| Transcendental future (TDL) |  | 0.376 | 2, 443 | .687 | .002 |
| Transcendent future (TDT) |  | 0.121 | 2, 443 | .886 | .001 |
| *Level of education* | | | | | |
| Multivariate test | .036 | 2.033 | 8, 882 | .040 | .018 |
| Positive future (PF) |  | 4.579 | 2, 443 | .011 | .020 |
| Negative future (NF) |  | 2.594 | 2, 443 | .076 | .012 |
| Transcendental future (TDL) |  | 0.205 | 2, 443 | .815 | .001 |
| Transcendent future (TDT) |  | 1.113 | 2, 443 | .330 | .005 |

*Note. N* = 446. Place of residence: rural (*n* = 121), town of fewer than 100,000 inhabitants (*n* = 149), city of 100,000 or more inhabitants (*n* = 176). Level of education: primary or vocational (*n* = 78), secondary (*n* = 208), higher (*n* = 160). Each variable was analysed in a separate one-way multivariate analysis of variance with the four future time perspective dimensions as dependent variables. Multivariate tests are based on Pillai’s trace, with *η*²*_p_* computed as *V*/*s*. Univariate tests are based on Type III sums of squares, with *η*²*_p_* computed as SS_effect_ / (SS_effect_ + SS_residual_). Place of residence had no multivariate effect on the four dimensions. Level of education showed a small but significant multivariate effect, and in the follow-up univariate tests the only significant effect was that on positive future; the corresponding descriptive statistics and post hoc comparisons are given in Table S8. Box’s *M* test was non-significant for both variables (place of residence: χ²(20) = 25.134, *p* = .196; level of education: χ²(20) = 17.960, *p* = .590), and in the analysis of level of education Levene’s test was non-significant for all four dependent variables.

**Table S8**

*Descriptive Statistics and Post Hoc Comparisons for the Four Future Time Perspective Dimensions by Level of Education*

| Descriptive statistics | | | | Post hoc comparisons (Tukey) | | | | |
| --- | --- | --- | --- | --- | --- | --- | --- | --- |
| Level of education | *n* | *M* | SD | Comparison | *ΔM* | SE | *t* | *p*_Tukey_ |
| *Positive future (PF)* | | | | | | | | |
| Primary/vocational | 78 | 11.47 | 1.63 | 1 vs. 2 | −0.04 | 0.24 | −0.15 | .988 |
| Secondary | 208 | 11.51 | 1.88 | 1 vs. 3 | −0.56 | 0.25 | −2.27 | .062 |
| Higher | 160 | 12.03 | 1.72 | 2 vs. 3 | −0.52 | 0.19 | −2.79 | .015 |
| *Negative future (NF)* | | | | | | | | |
| Primary/vocational | 78 | 15.55 | 4.71 | 1 vs. 2 | −0.62 | 0.76 | −0.82 | .693 |
| Secondary | 208 | 16.17 | 5.81 | 1 vs. 3 | 0.75 | 0.79 | 0.95 | .610 |
| Higher | 160 | 14.81 | 5.96 | 2 vs. 3 | 1.36 | 0.60 | 2.28 | .060 |
| *Transcendental future (TDL)* | | | | | | | | |
| Primary/vocational | 78 | 23.06 | 7.24 | 1 vs. 2 | 0.05 | 0.96 | 0.05 | .999 |
| Secondary | 208 | 23.02 | 7.07 | 1 vs. 3 | −0.42 | 1.00 | −0.42 | .906 |
| Higher | 160 | 23.49 | 7.45 | 2 vs. 3 | −0.47 | 0.76 | −0.62 | .812 |
| *Transcendent future (TDT)* | | | | | | | | |
| Primary/vocational | 78 | 32.71 | 5.76 | 1 vs. 2 | −0.58 | 0.83 | −0.70 | .763 |
| Secondary | 208 | 33.29 | 6.15 | 1 vs. 3 | −1.24 | 0.87 | −1.43 | .326 |
| Higher | 160 | 33.94 | 6.64 | 2 vs. 3 | −0.66 | 0.66 | −1.00 | .581 |

*Note. N* = 446. Groups are numbered 1 = primary or vocational education, 2 = secondary education, 3 = higher education. *ΔM* is the mean difference between the two groups indicated, expressed in raw scale points, and *p*_Tukey_ is the probability adjusted for comparing a family of three means. The only significant contrast was that between secondary and higher education on positive future; participants with higher education reported a slightly more positive view of their personal future. Level of education was unrelated to the remaining three dimensions.

**Table S9**

*Education-Adjusted Sensitivity Specification of Model A Predicting the Four Future Time Perspective Dimensions*

| Predictor | *B* | SE | *z* | *p* | *β* | 95% CI for B |
| --- | --- | --- | --- | --- | --- | --- |
| *Positive future (PF)* | | | | | | |
| GM | 0.021 | 0.009 | 2.309 | .021 | .105 | [0.003, 0.038] |
| BLD | 0.025 | 0.012 | 2.115 | .034 | .099 | [0.002, 0.049] |
| Age | −0.003 | 0.014 | −0.242 | .809 | −.012 | [−0.032, 0.025] |
| Primary/vocational | −0.044 | 0.234 | −0.190 | .849 | −.009 | [−0.504, 0.415] |
| Higher | 0.529 | 0.191 | 2.773 | .006 | .142 | [0.155, 0.903] |
| *Negative future (NF)* | | | | | | |
| GM | −0.235 | 0.026 | −9.122 | < .001 | −.373 | [−0.286, −0.185] |
| CD | 0.242 | 0.042 | 5.735 | < .001 | .230 | [0.159, 0.324] |
| FD | 0.217 | 0.040 | 5.353 | < .001 | .220 | [0.137, 0.296] |
| Age | 0.021 | 0.040 | 0.528 | .597 | .022 | [−0.057, 0.099] |
| Primary/vocational | −0.467 | 0.643 | −0.727 | .467 | −.031 | [−1.727, 0.793] |
| Higher | −1.191 | 0.525 | −2.270 | .023 | −.100 | [−2.220, −0.163] |
| *Transcendental future (TDL)* | | | | | | |
| BLD | 0.790 | 0.028 | 28.257 | < .001 | .762 | [0.735, 0.845] |
| CD | 0.244 | 0.036 | 6.845 | < .001 | .184 | [0.174, 0.313] |
| FD | 0.079 | 0.026 | 3.058 | .002 | .063 | [0.028, 0.129] |
| Age | 0.015 | 0.030 | 0.494 | .621 | .013 | [−0.044, 0.074] |
| Primary/vocational | 0.407 | 0.486 | 0.837 | .403 | .021 | [−0.546, 1.359] |
| Higher | 0.295 | 0.396 | 0.744 | .457 | .020 | [−0.482, 1.072] |
| *Transcendent future (TDT)* | | | | | | |
| BLD | 0.309 | 0.038 | 8.136 | < .001 | .343 | [0.234, 0.383] |
| CD | 0.400 | 0.048 | 8.298 | < .001 | .349 | [0.305, 0.494] |
| Age | 0.040 | 0.041 | 0.971 | .331 | .040 | [−0.041, 0.122] |
| Primary/vocational | −0.503 | 0.673 | −0.747 | .455 | −.031 | [−1.823, 0.817] |
| Higher | 0.502 | 0.549 | 0.916 | .360 | .039 | [−0.573, 1.578] |

*Note. N* = 446. Level of education was represented by two dummy variables, with secondary education as the reference category: primary/vocational (1 = primary or vocational education, 0 = otherwise) and higher (1 = higher education, 0 = otherwise). *B* = unstandardized regression coefficient; *SE* = standard error; *β* = fully standardized estimate; CI = confidence interval for *B*. This sensitivity specification retained the residual covariances among PF, NF, TDL, and TDT estimated in Model A. Model fit: χ²(6) = 8.554, *p* = .200, CFI = .998, TLI = .987, RMSEA = .031, 90% CI [.000, .074], SRMR = .013. Explained variance: *R²* = .041 (PF), .297 (NF), .746 (TDL), .350 (TDT), compared with *R²* = .022 (PF), .289 (NF), .746 (TDL), .348 (TDT) in Model A. Fit was thus equivalent to that of Model A (see Table S4). Because the two specifications involve different sets of observed variables, they are not nested and no chi-square difference test is reported. Two of the eight education paths reached significance: relative to secondary education, higher education was associated with higher positive future and with lower negative future. The latter path emerges once general mood, contemplation of death, fear of death, and age are held constant, and is therefore not reflected in the unadjusted univariate comparison reported in Table S7. All remaining paths, including those predicting positive future from general mood and belief in life after death, were substantively unchanged relative to Model A.

**Table S10**

*Multivariate Analysis of Variance for the Four Future Time Perspective Dimensions (Pillai’s Trace)*

| Effect | Pillai’s V | Approx. F | df1 | df2 | p | Wilks’ Λ |
| --- | --- | --- | --- | --- | --- | --- |
| General mood | .160 | 20.761 | 4 | 435 | < .001 | .840 |
| Gender | .066 | 7.682 | 4 | 435 | < .001 | .934 |
| Age group | .025 | 2.827 | 4 | 435 | .025 | .975 |
| General mood × Gender | .007 | 0.742 | 4 | 435 | .564 | .993 |
| General mood × Age group | .006 | 0.683 | 4 | 435 | .604 | .994 |
| Gender × Age group | .015 | 1.660 | 4 | 435 | .158 | .985 |
| General mood × Gender × Age group | .026 | 2.906 | 4 | 435 | .021 | .974 |

*Note.* N = 446; residual df = 438. General mood was standardized and dichotomized at z = 0 (lower mood: n = 192; higher mood: n = 254); age group was 60–70 years (n = 249) versus 71–95 years (n = 197); gender was female (n = 226) versus male (n = 220). There were no missing data on any factor. Assumption checks: Box’s M-test indicated a marginal departure from homogeneity of the covariance matrices, χ²(70) = 91.856, p = .041, and the Shapiro–Wilk test indicated a departure from multivariate normality, W = 0.987, p < .001. Because cell sizes were moderately large and approximately balanced (n = 44 to 74) and because Pillai’s trace is the most robust of the multivariate criteria under such violations, Pillai’s trace is reported as the primary test; Wilks’ Λ is reported for completeness and yields identical F values and p values. Levene’s test is not applicable to the multivariate design and is therefore not reported.

**Table S11**

*Univariate Analyses of Variance for Each Future Time Perspective Dimension*

| Effect | SS | df | MS | F | p | η² | |  |
| --- | --- | --- | --- | --- | --- | --- | --- | --- |
| **Positive future (PF)** | | | | | | | |  |
| Mood | 38.155 | 1 | 38.155 | 12.468 | < .001 | .028 | |  |
| Gender | 18.457 | 1 | 18.457 | 6.032 | .014 | .014 | |  |
| Age | 4.600 | 1 | 4.600 | 1.503 | .221 | .003 | |  |
| Mood × Gender | 7.589 | 1 | 7.589 | 2.480 | .116 | .006 | |  |
| Mood × Age | 5.322 | 1 | 5.322 | 1.739 | .188 | .004 | |  |
| Gender × Age | 14.823 | 1 | 14.823 | 4.844 | .028 | .011 | |  |
| Mood × Gender × Age | 4.009 | 1 | 4.009 | 1.310 | .253 | .003 | |  |
| Residuals | 1,340.344 | 438 | 3.060 |  |  |  | |  |
| **Negative future (NF)** | | | | | | | |  |
| Mood | 1,677.036 | 1 | 1,677.036 | 60.720 | < .001 | .122 | |  |
| Gender | 375.881 | 1 | 375.881 | 13.609 | < .001 | .030 | |  |
| Age | 25.401 | 1 | 25.401 | 0.920 | .338 | .002 | |  |
| Mood × Gender | 0.130 | 1 | 0.130 | 0.005 | .945 | .000 | |  |
| Mood × Age | 24.585 | 1 | 24.585 | 0.890 | .346 | .002 | |  |
| Gender × Age | 7.595 | 1 | 7.595 | 0.275 | .600 | .001 | |  |
| Mood × Gender × Age | 287.283 | 1 | 287.283 | 10.401 | .001 | .023 | |  |
| Residuals | 12,097.294 | 438 | 27.619 |  |  |  | |  |
| **Transcendental future (TDL)** | | | | | | | |  |
| Mood | 0.653 | 1 | 0.653 | 0.013 | .909 | .000 | |  |
| Gender | 917.311 | 1 | 917.311 | 18.189 | < .001 | .040 | |  |
| Age | 172.810 | 1 | 172.810 | 3.427 | .065 | .008 | |  |
| Mood × Gender | 7.802 | 1 | 7.802 | 0.155 | .694 | .000 | |  |
| Mood × Age | 0.143 | 1 | 0.143 | 0.003 | .958 | .000 | |  |
| Gender × Age | 0.735 | 1 | 0.735 | 0.015 | .904 | .000 | |  |
| Mood × Gender × Age | 21.813 | 1 | 21.813 | 0.433 | .511 | .001 | |  |
| Residuals | 22,088.762 | 438 | 50.431 |  |  |  | |  |
| **Transcendent future (TDT)** | | | | | | | |  |
| Mood | 96.896 | 1 | 96.896 | 2.522 | .113 | .006 | |  |
| Gender | 443.710 | 1 | 443.710 | 11.549 | < .001 | .026 | |  |
| Age | 10.496 | 1 | 10.496 | 0.273 | .601 | .001 | |  |
| Mood × Gender | 2.283 | 1 | 2.283 | 0.059 | .808 | .000 | |  |
| Mood × Age | 4.389 | 1 | 4.389 | 0.114 | .736 | .000 | |  |
| Gender × Age | 67.822 | 1 | 67.822 | 1.765 | .185 | .004 | |  |
| Mood × Gender × Age | 22.858 | 1 | 22.858 | 0.595 | .441 | .001 | |  |
| Residuals | 16,828.298 | 438 | 38.421 |  |  |  | |  |

*Note.* N = 446; df = 1, 438 for every effect. Mood = general mood (standardized and dichotomized at z = 0); Age = age group (60–70 vs. 71–95 years). Type III sums of squares. η² was computed as SS_effect_ / (SS_effect_ + SS_residual_), that is, as partial η². Cell means for the two significant interaction effects are given in Table S12 and displayed in Figures S2 and S3. Tests of simple effects for these two interactions are reported in Table S13.

**Table S12**

*Cell Means for the Significant Interaction Effects*

| General mood | Gender | Age group | n | M | SD | SE | |  |
| --- | --- | --- | --- | --- | --- | --- | --- | --- |
| **Positive future: Gender × Age group** | | | | | | | |  |
| — | Women | 60–70 | 130 | 11.946 | 1.690 | 0.148 | |  |
| — | Women | 71–95 | 96 | 11.781 | 1.766 | 0.180 | |  |
| — | Men | 60–70 | 119 | 11.252 | 1.805 | 0.165 | |  |
| — | Men | 71–95 | 101 | 11.792 | 1.873 | 0.186 | |  |
| **Negative future: General mood × Gender × Age group** | | | | | | | |  |
| Low | Women | 60–70 | 59 | 19.966 | 5.021 | 0.654 | |  |
| Low | Women | 71–95 | 44 | 16.955 | 4.754 | 0.717 | |  |
| Low | Men | 60–70 | 45 | 16.178 | 4.438 | 0.662 | |  |
| Low | Men | 71–95 | 44 | 17.409 | 4.838 | 0.729 | |  |
| High | Women | 60–70 | 71 | 14.338 | 4.934 | 0.586 | |  |
| High | Women | 71–95 | 52 | 15.462 | 5.812 | 0.806 | |  |
| High | Men | 60–70 | 74 | 13.527 | 5.871 | 0.683 | |  |
| High | Men | 71–95 | 57 | 12.351 | 5.743 | 0.761 | |  |

*Note.* N = 446. Means for the Gender × Age group interaction on positive future are collapsed across general mood. The Gender × Age group interaction on positive future reflects convergence rather than a male advantage: men in the older group scored slightly higher than in the younger group whereas women showed a small decline, with the result that the two groups do not differ meaningfully after age 70 (M = 11.79 for men and M = 11.78 for women).

**Table S13**

*Simple Effects Analyses for the Two Significant Interaction Effects*

| Gender | Age group | *F* | *df* | *p* | η²_p_ |
| --- | --- | --- | --- | --- | --- |
| *Negative future: simple effect of general mood* | | | | | |
| Women | 60–70 | 36.96 | 1, 438 | < .001 | .078 |
| Women | 71–95 | 1.92 | 1, 438 | .166 | .004 |
| Men | 60–70 | 7.12 | 1, 438 | .008 | .016 |
| Men | 71–95 | 23.00 | 1, 438 | < .001 | .050 |
| *Positive future: simple effect of gender* | | | | | |
| — | 60–70 | 9.45 | 1, 442 | .002 | .021 |
| — | 71–95 | 0.00 | 1, 442 | .966 | < .001 |

*Note. N* = 446. Simple effects were tested within the corresponding factorial model using the pooled error term. For negative future, the simple effect of general mood (lower vs. higher) was tested at each combination of gender and age group within the full Gender × Age group × General mood model (MS_residual_ = 27.62). For positive future, the simple effect of gender was tested at each level of age group within the corresponding Gender × Age group model (MS_residual_ = 3.17), which accounts for the different error degrees of freedom. η²_p_ was computed as SS_effect_ / (SS_effect_ + SS_residual_). Cell means and standard deviations for all cells are reported in Table S12 and displayed in Figures S2 and S3.

**Figure S1**

*Path Diagram for Model A With Standardized Estimates*

*Note.* N = 446. Values are fully standardized regression coefficients. Solid arrows denote statistically significant paths and dashed grey arrows denote non-significant paths. The six residual covariances among the four outcome variables were freely estimated but are not displayed; they are reported in Table S3. * p < .05. ** p < .01. *** p < .001.

**Figure S2**

*Gender × Age Group Interaction for Positive Future*

*Note.* N = 446. Error bars represent ±1 standard error. The interaction was significant in the full three-way design, F(1, 438) = 4.844, p = .028, partial η² = .011. Cell sizes were 130, 96, 119, and 101. The pattern reflects convergence of the two gender groups after age 70 rather than higher scores among older men.

**Figure S3**

*Gender × Age Group × General Mood Interaction for Negative Future*

*Note.* N = 446. Error bars represent ±1 standard error. The three-way interaction was significant, F(1, 438) = 10.401, p = .001, partial η² = .023. Cell sizes ranged from 44 to 74. Among participants with low general mood, negative future declined with age in women but increased slightly in men; among participants with high general mood the pattern was reversed.
